# A Practical Review of NMR Lineshapes for Spin-1/2 and Quadrupolar Nuclei in Disordered Materials

**DOI:** 10.3390/ijms21165666

**Published:** 2020-08-07

**Authors:** Kuizhi Chen

**Affiliations:** Nation High Magnetic Field Laboratory, Tallahassee, FL 32310, USA; kuizhi.chen@magnet.fsu.edu

**Keywords:** disorder, solid-state NMR, amorphous material, quadrupolar nuclei, lineshape, high-field NMR, DQ–SQ, MQMAS, inhomogeneous broadening

## Abstract

NMR is a powerful spectroscopic method that can provide information on the structural disorder in solids, complementing scattering and diffraction techniques. The structural disorder in solids can generate a dispersion of local magnetic and electric fields, resulting in a distribution of isotropic chemical shift δ_iso_ and quadrupolar coupling C_Q_. For spin-1/2 nuclei, the NMR linewidth and shape under high-resolution magic-angle spinning (MAS) reflects the distributions of isotropic chemical shift, providing a rich source of disorder information. For quadrupolar nuclei, the second-order quadrupolar broadening remains present even under MAS. In addition to isotropic chemical shift, structural disorder can impact the electric field gradient (EFG) and consequently the quadrupolar NMR parameters. The distributions of quadrupolar coupling and isotropic chemical shift are superimposed with the second-order quadrupolar broadening, but can be potentially characterized by MQMAS (multiple-quantum magic-angle spinning) spectroscopy. We review analyses of NMR lineshapes in 2D DQ–SQ (double-quantum single-quantum) and MQMAS spectroscopies, to provide a guide for more general lineshape analysis. In addition, methods to enhance the spectral resolution and sensitivity for quadrupolar nuclei are discussed, including NMR pulse techniques and the application of high magnetic fields. The role of magnetic field strength and its impact on the strategy of determining optimum NMR methods for disorder characterization are also discussed.

## 1. Introduction

Disordered structures are found in a wide variety of solid-state materials, such as glasses [1,2], polymers [3,4], batteries [5,6], solid state catalysts [7,8], metal-organic frameworks [9,10], etc. Many of these materials have partial or complete crystallinity, and are therefore typically characterized using scattering and diffraction techniques. However, these techniques offer only “average” structural information [11]. Solid state NMR is a complementary method that can provide local structural information at the atomic level without the requirement of the long-range order and periodicity. Material structures can be characterized in two different regimes in solid-state NMR, namely, spectroscopy [2,12,13] and relaxometry [3,4,14]. In particular, the former refers to the spectral analysis such as chemicals shift, lineshape and linewidth, while the latter consists of measurements of nuclear relaxation times [14]. The focus of this review will be on the “spectroscopy” regime in solid-state NMR. We present a survey of modern NMR techniques, which can serve as a useful summary for experts in the field, but is primarily intended as a practical introduction for material scientists whose research may benefit from these cutting-edge applications of NMR.

The local magnetic field induced by the external magnetic field, i.e., the chemical shielding, is reflected as a chemical shift and is sensitive to the surrounding electron environment of the nuclei. A distinct chemical shift stemmed from the variety chemical bondings provides the NMR spectral resolution and assignment. A structural disorder can cause small changes to the local environment, which can lead to variations of the chemical shielding. For spin-1/2 nuclei, magic-angle spinning (MAS) averages chemical shift anisotropy (CSA) yielding high-resolution spectra with only the isotropic chemical shift δ_iso_. Thus, the line broadening and shape in MAS spectra of spin-1/2 nuclei reflect directly the distribution of δ_iso_ induced by the disorder. For spin>1/2 nuclei, an additional spin interaction can occur between the electric quadrupole moment and the electric field gradient (EFG) at the nuclear site. The quadrupole moment is zero for all spin-1/2 nuclei, whereas spin>1/2 nuclei can have quadrupolar interactions, and are therefore referred to as quadrupolar nuclei. Large quadrupolar interactions cause rapid spin-spin (*T*_2_) relaxation and significant quadrupolar broadening, making NMR spectroscopy of quadrupolar nuclei relatively more difficult than spin-1/2 nuclei. A structural disorder affects the NMR spectra of quadrupolar nuclei through both the δ_iso_ and the EFG, which can be represented by C_Q_ (quadrupolar coupling constant) and η_Q_ (asymmetry parameter). Thus, the characterization of quadrupolar nuclei is more complicated due to the superimposition of isotropic and quadrupolar interactions. However, given that the quadrupolar interaction provides an additional “set of parameters” that can reflect the local structural changes, performing NMR characterizations on quadrupolar nuclei in addition to more common spin-1/2 nuclei could play important roles in revealing material structures.

In the periodic table, more than 75% of NMR active nuclei are quadrupolar and many of them such as ^27^Al, ^11^B, ^71^Ga, ^73^Ge, ^17^O, ^23^Na and ^67^Zn can be found in various type of materials. These nuclei have half-integer spins (I = 3/2, 5/2, 7/2 and 9/2) and the quadrupolar interaction to the central m = 1/2 <-> -1/2 transition (CT) vanishes at the first-order. Compared to the broad signals arising from satellite transitions (STs), relatively narrow CT signals can be acquired for these half-integer quadrupolar nuclei. However, the second-order quadrupolar interaction still remains even under magic-angle spinning (MAS). Therefore, MAS NMR spectra, which display both an isotropic chemical shift and a second-order quadrupolar interaction, are more complex than spin-1/2 nuclei. Note that the latter consists of both the isotropic quadrupolar shift and anisotropic broadening terms. Several methods have been developed to remove the quadrupolar broadening, namely, dynamic angle spinning (DAS) [15,16], double rotation (DOR) NMR [15,16], multiple-quantum magic-angle spinning (MQMAS) [17,18] and satellite transition magic-angle spinning (STMAS) [19,20]. Two-dimensional MQMAS and STMAS have gained popularity recently as they can refocus the anisotropic quadrupolar broadening using standard MAS probes. The separation between isotropic and quadrupolar terms can be achieved by these two methods as the relative scales between isotropic chemical and quadrupolar shifts varies between the different transitions presented along the two dimensions [21]. These 2D experiments can help to untangle various contributions to the spectral broadening from structural disorder.

High magnetic fields are of particular importance to solid-state NMR of quadrupolar nuclei. The application of high fields can provide direct improvement to the spectral resolution in 1D MAS spectra through the reduction of anisotropic second-order quadrupolar broadening that remains under MAS. This high-field advantage has been demonstrated using low-homogeneity unregulated superconducting/resistive hybrid magnet up to 40 T at the National High Magnetic Field Laboratory (NHMFL) [22]. The NHMFL has recently commissioned a series-connected-hybrid (SCH) magnet with a more homogeneous and regulated field up to 36 T [23]. The availability of high-field superconducting magnets and the even higher hybrid magnets is particularly useful to enhance the spectral resolution and sensitivity of quadrupolar nuclei for both 1D and more demanding 2D experiments, as well as for highly crystalline and amorphous solids with a disorder. We will provide a detailed discussion of the 2D MQMAS method with high-fields in a later section. Typically, NMR experiments performed on quadrupolar nuclei have low sensitivity and/or broad-line problems due to low gyromagnetic ratios, low natural abundances and/or large quadrupole moments. This review will give a brief overview of modern NMR methods, including some from recent developments that address these issues related to quadrupolar nuclei, such as QCPMG (quadrupolar Carr–Purcell–Meiboom–Gill) [24,25] and central transition polarization [26,27], which enhance sensitivity, and QMAT (quadrupolar magic angle turning)/QPASS (quadrupolar phase adjusted sideband suppression), which separate isotropic band from sidebands [28,29,30].

In this review, we demonstrate several techniques of disorder characterization in both the spin-1/2 and quadrupolar nuclei cases, taking solid catalyst zeolite, among a few other materials from the literature, as our primary examples. Zeolite is usually known as a crystalline material with microporous framework structure, but the location of active sites and amorphous protonic contents can be considered as disordered on the environments to the nuclei under investigation. In addition, the existence of numerous distinct crystalline inequivalent sites, for example as many as 12 in the case of MFI zeolites [31,32], can have similar effects and consequences to ^27^Al and ^17^O NMR [8,33,34], when not resolved, as distributions of isotropic chemical shift and quadrupolar coupling parameters from disordered structures. DFT (Density Functional Theory) calculations have been used to directly relate NMR parameters to structural differences and variations, modeled either as many distinct crystalline sites or continuous distributions from disorder. [11] Such an approach has prompted increased interest in recent research in heterogeneous catalysis, such as revealing aluminum/proton site distributions in zeolite [32,35,36] and amorphous silica-alumina catalysts [37].

The following discussion is divided into two sections. Section 2 focuses on revisiting the basics of NMR lineshapes for spin-1/2 nuclei in both 1D and 2D spectroscopy, followed by an illustration of the effects of disorder on the lineshape, with examples on 2D DQ–SQ correlation spectroscopy and the various interpretation methods from the literature. Section 3 extends the discussion of disorder effects to quadrupolar NMR nuclei, by first reviewing the fundamentals of quadrupolar NMR spectroscopy, including high-field effects, and then detailing the strategy of disorder characterization through examples of MQMAS experiments.

## 2. NMR Detection of Spin-1/2 Nuclei in Disordered Environments

### 2.1. Impacts of Disorder on 1D NMR Spectroscopy

#### 2.1.1. NMR Lineshapes of Spin-1/2 Nuclei

The Fourier transformed NMR spectrum of an isolated I = 1/2 spin can be represented mathematically by Equation 1, where Ω_0_ is the center frequency of the peak, S_0_ is the signal intensity and R = T_2_^−1^ is the transverse relaxation rate. Defined by the equation, the peak is a Lorentzian shape [38].

(1) $$S\left(\Omega \right)=\frac{S_{0}R}{{\left(\Omega -\Omega_{0}\right)}^{2}+R^{2}}$$

For an ensemble of spins with a distribution of its resonant frequency, the lineshape can become Gaussian or a mixture of both components. The Lorentzian lineshape is often found in the spectra of solutions, while the Gaussian shape is more common in solids as a result of a distribution of Lorentzian peaks due to the rigidity of the system [39]. The NMR peak can be broadened by two mechanisms, namely, homogeneous and heterogeneous broadening, which stem from two different types of local field interactions. Briefly, homogeneous broadening is generated by randomly fluctuating local fields, whereas the inhomogeneous broadening arises from the local field that does not vary in the time scale of the signal measurement [40,41]. The disorder in materials, as the primary interest in this review, can often lead to inhomogeneous broadening. Although the inhomogeneous broadening can arise from multiple factors as listed below, the essential origins can all be attributed to a distribution of the resonant frequency induced by the magnetic field dispersion. It is important to inspect the factors causing the spectral broadening for the purpose of disorder characterizations. Including structural disorder of the material, a few common factors that result in inhomogeneous broadening and their origins can be summarized as follows:

- Structural disorder, chemical shielding;
- CSA (chemical shift anisotropy), chemical shielding;
- Imperfect shimming, external field;
- Bulk magnetic susceptibility (BMS), internal field;
- Anisotropy bulk magnetic susceptibility (ABMS), internal field;
- Dipolar coupling from nearby spins, dipole–dipole interaction;
- J-coupling, through-bond interaction.

Although many factors exist, the interesting term, i.e., the disorder induced broadening (**a**), can still be safely characterized with common NMR techniques employed, particularly, MAS (magic-angle spinning) and decoupling methods, as the disorder broadening is not affected by either of them. Among the factors, the CSA (**b**) and dipolar coupling (**f**) are the most prominent interactions but can be sufficiently suppressed by MAS [40]. In general, the magnitude of CSA ranges from tens of ppm in ^1^H to 120–140 ppm in ^13^C and can be much larger in some heavier nuclei (up to 1000 s ppm) [42]. It is important to notice that the magnitude of CSA is constant in ppm at variable fields. In contrast, dipolar coupling is constant in Hz at variable fields with magnitude ranges from a few to tens of kHz (up to 100 kHz between two proton spins), depending on the gyromagnetic ratios and spatial distances between the coupled spins [38,42]. In addition to MAS, hetero- [43] and homo-nuclear [44,45] decoupling methods have been extensively used to reduce or remove the dipolar coupling broadening. The other interactions are minor but worthy of inspections in order to ensure that disorder is the primary effect contributing to the lineshapes being analyzed. Imperfect shimming (**c**) creates an external field gradient that broadens the peak, and thus its effect cannot be removed by MAS. The bulk magnetic susceptibility (BMS) (**d**) refers to magnetic fields induced at different parts of the sample, such as the field induced by bubbles in liquid and voids in solid samples, whose susceptibility tensor is NOT orientation dependent, and therefore behaves like magnetic dipoles and can be removed by MAS [46]. The anisotropic bulk magnetic susceptibility (ABMS) (**e**)**,** on the order of 1–2 ppm, produces a dispersion of chemical shifts in a way similar to the ring current shifts in aromatic systems, and therefore cannot be removed by MAS [47,48]. Hence, ABMS needs to be treated carefully in the lineshape analysis of disordered structures. Finally, the through-bond J-coupling (**g**) is not removed by MAS but is usually considered a weak interaction, as it is normally <200 Hz, e.g., 135 Hz for one-bond C-H J-coupling [38]. Therefore, the minor interactions (**c**), (**d**), (**e**) and (**g**) can often be neglected in the lineshape analysis in solids.

#### 2.1.2. Removal of CSA and Dipolar Coupling by MAS

**Figure 1:**
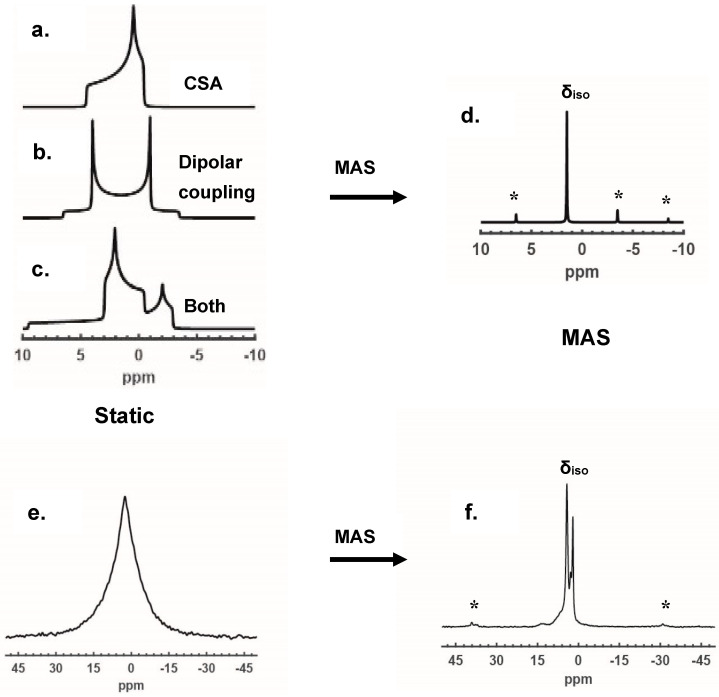
(**a**–**c**) Simulated powder lineshapes of static spectra (^13^C at 9.4 T) with 3 kHz chemical shift anisotropy (CSA) and 5 kHz dipolar coupling interactions introduced separately or together as indicated in the figures. With 5 kHz magic-angle spinning, all inhomogeneous patterns in (**a**–**c**) break up to an isotropic peak and spinning sidebands, as shown in (**d**). (**e**,**f**) show the suppression of dipolar coupling and CSA for a dehydrated zeolite HZSM-5 catalyst in ^1^H NMR by magic-angle spinning (MAS). The spectra of (**e**,**f**) were acquired at 9.4 T, where (**f**) was acquired at spinning frequency of 10 kHz. Spinning sidebands are denoted in “*”.

Magic-angle spinning (MAS) is the technique where the sample is packed in a rotor (or a holder) and rotated at the ‘magic’ angle of θ = 54.74° to the *z*-axis. Such a rotation is sufficient to average out the removable inhomogeneous interactions listed above. CSA (chemical shift anisotropy) and dipolar coupling are the most common interactions and their removals by MAS are illustrated in Figure 1 in both simulated and experimental spectra. In a powder crystalline sample, CSA and dipolar coupling can create an orientation-dependent local field for spins in each crystallite via chemical shielding and dipole–dipole interaction, respectively. Each crystallite will result in a single NMR resonance. However, the superposition of the individual spectrum of each randomly oriented crystallite is observed as an overall spectrum with powder patterns. Figure 1a,b demonstrates anisotropic powder patterns of ^13^C spectra at 9.4 T with 3 kHz of CSA and 5 kHz of dipolar coupling interactions, respectively. The two interactions can also coexist in the same sample, as illustrated in Figure 1c. With 5 kHz MAS applied, CSA and dipolar couplings both vanished, leaving a resolved isotropic peak δ_iso_ with spinning sidebands separated by the spinning frequency, as shown in Figure 1d. Hence, the MAS condition is normally required for disorder characterizations. An experimental example illustrating the MAS effects on CSA and dipolar coupling is shown in Figure 1e,f, by comparing the static and MAS ^1^H NMR spectra of a dehydrated HZSM-5 catalyst acquired at 9.4 T. At the static condition, the spectrum appeared as one broad peak with a full-width-at-half-maximum (FWHM) around 5 kHz, dominated by dipolar coupling and CSA interactions, which make the spectral analysis difficult. However, with 10 kHz MAS, both dipolar coupling and CSA are sufficiently averaged out, leaving distinct proton species resolved in narrow isotropic peaks for further analysis. It is worth noting that dipolar interactions among abundant spins with high gyromagnetic ratios such as ^1^H and ^19^F may lead to homonuclear broadening even under MAS [41]. However, such broadening can be reduced by increasing spinning speed, increasing field strength and/or employing decoupling sequences [41,44]. In this case, because the zeolite catalyst possesses a dilute proton system, the homonuclear-coupling-induced broadening is not a concern at moderate spinning speed (10 kHz).

#### 2.1.3. Effect of Disorder on 1D MAS NMR Spectroscopy

**Figure 2:**
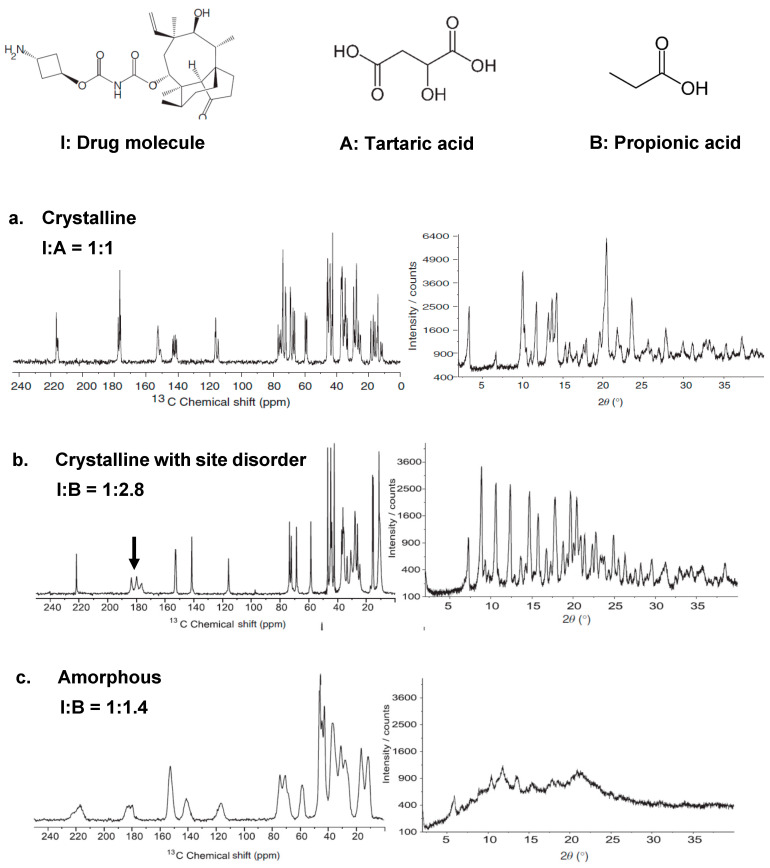
^13^C CP-MAS spectra and XRD patterns showing a drug material **I** prepared with different degrees of disorder. **A** and **B** are both base molecules that can affect the formation of crystallinity for **I**. The material in crystalline (**a**), crystalline with site disorder (**b**) and amorphous (**c**) forms were prepared by mixing **I** with **A** or **B** at ratios indicated in each figure. Reprinted from Ref. [12], Copyright (2011), with permission from Elsevier.

Chemical shift is very sensitive to the local environment of the nucleus. The changes in the chemical shift can essentially be attributed to deviations of the local magnetic fields, which, are induced by changes in chemical shielding and can be generated by a number of possible factors including the structural disorder, molecules with different conformations, a rich hydrogen bonding environment, or imperfect crystalline packings, among others. Significant changes of local environment (i.e., changes in coordination number) will lead to large changes of the chemical shift, observed as separated peaks. Relatively small changes (i.e., changes in bond angle and/or bond distance) will result in variations to the chemical shift, leading to a chemical shift distribution, observed as inhomogeneous broadening to the spectrum. Such a broadening effect is illustrated in Figure 2 by an NMR investigation of stepwise morphology change (“crystalline” to “crystalline with site disorder” to “amorphous”) in a drug molecule system, adapted from work by Clawson et al. [12]. Figure 2a shows a ^13^C CP MAS spectrum of a crystalline powder sample formed by mixing the drug molecule **I** in base **A** (tartaric acid) in a 1: 1 ratio, where the molecular structures are illustrated on top of the figure. The sharp lines in the NMR spectrum suggest that the system attains a high crystallinity, in agreement with the sharp diffraction peaks in the XRD pattern. In Figure 2b, by crystallizing the mixture of **I** and base **B** in a 1: 2.8 ratio, a disorder occurred selectively on a carbonyl site, visualized as broadening effects on the carbonyl peaks at around 180 ppm. The triplet is indicative of a crystalline system with multiple molecules in the asymmetric unit (*Z*’), according to the author. The XRD pattern, however, still shows high crystallinity due to the lack of sensitivity to the local site disorder. When the sample was prepared in a non-crystalline form by mixing **I** and base **B** in a specific ratio 1: 1.4, as indicated in Figure 2c, both the NMR and XRD pattern were broadened and lost resolution, due to the loss of long-range order. Note that the inhomogeneous broadening arising from the chemical shift distribution should always be differentiated from the homonuclear broadening [49]. One-dimensional spectroscopy is usually incapable of distinguishing between these two effects, but 2D NMR methods can, by producing different types of cross peaks in each case, as will be discussed below.

### 2.2. Impacts of Disorder on 2D NMR Spectroscopy

#### 2.2.1. Lineshapes in 2D NMR

**Figure 3:**
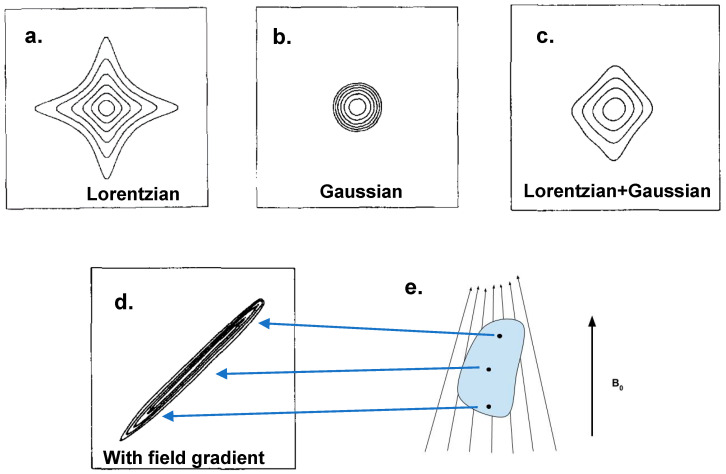
The line-shape of cross peaks in 2D spectroscopy in (**a**) Lorentzian, (**b**) Gaussian and (**c**) mixed shapes. The spectrum in (**a**,**b**) are simulated and in (**c**) is a ^23^Na NOESY spectrum acquired on 2 M NaCl in D_2_O. (**d**) is acquired on the same sample as of (**c**) but with a shim gradient applied during the experiment. The schematic in (**e**) illustrates three spins located at different positions of the sample and their frequencies in the spectrum. (**a**–**d**) are reprinted from Ref. [50], Copyright (1995), with permission from Elsevier.

It is well known that Lorentzian and Gaussian peaks appear in different shapes in 2D NMR, i.e., “star” and “round” shapes in each case, as illustrated in simulations in Figure 3a,b [50]. In solids, peak shapes are commonly Gaussian, or a mixture of both Gaussian and Lorentzian, in which case one expects a shape similar to those shown in Figure 3b,c. However, chemical shift dispersion, essentially caused by a dispersion of the local magnetic field, can “stretch” a 2D cross peak from a symmetric shape into an elongated ridge. The effect is illustrated here analogously by observing the shape changes on a solution 2D cross peak with a “field dispersion” intentionally introduced by shimming [50]. The Gaussian–Lorentzian mixed cross peak in Figure 3c was a 2D ^23^Na NOESY spectrum acquired on 2 M NaCl in D_2_O. Upon the introduction of the shimming gradient, the cross peak became a narrow ridge stretched along the diagonal, as shown in Figure 3d. The change occurred because atoms in different parts of the sample started to experience different effective magnetic fields, resulting in a distribution of resonant frequencies, which is visualized as a distribution of chemical shift. In other words, the ridge in Figure 3d is a superposition of cross peaks at different parts of the sample, as illustrated by the schematic in Figure 3e. In this specific case, the local field dispersion is manipulated by a shimming gradient in a static solution spectrum. However, the same effect occurs for isotropic peaks in solids at the MAS condition, as long as the local field dispersion exists. Indeed, the dispersion in solids can be caused by disorder but also ABMS as both of them are not removed by MAS. In a 1D spectrum, if the broadening is a homogeneous broadening, the broadened line is not a superposition of individual narrower peaks, and therefore the cross peak does not appear stretched but rather as a more circular shape [51].

#### 2.2.2. Effect of the Disorder on 2D MAS NMR Spectroscopy

The lineshape information contained in the 2D spectrum can be extremely useful for understanding the structural disorder. Promising applications have been reported in the characterization of disordered materials using 2D homonuclear or heteronuclear correlation experiments, for example, double-quantum single-quantum (DQ–SQ) [13,51] and heteronuclear correlation (HETCOR) experiments [48,52]. These methods normally show remarkable resolution improvements compared to 1D spectroscopy because of the presence of the correlated connections between species and the absence of signal for non-coupled spins. Although only DQ–SQ spectroscopy was chosen for lineshape analysis in this review, by no means is it the only method for this type of analysis.

**Figure 4:**
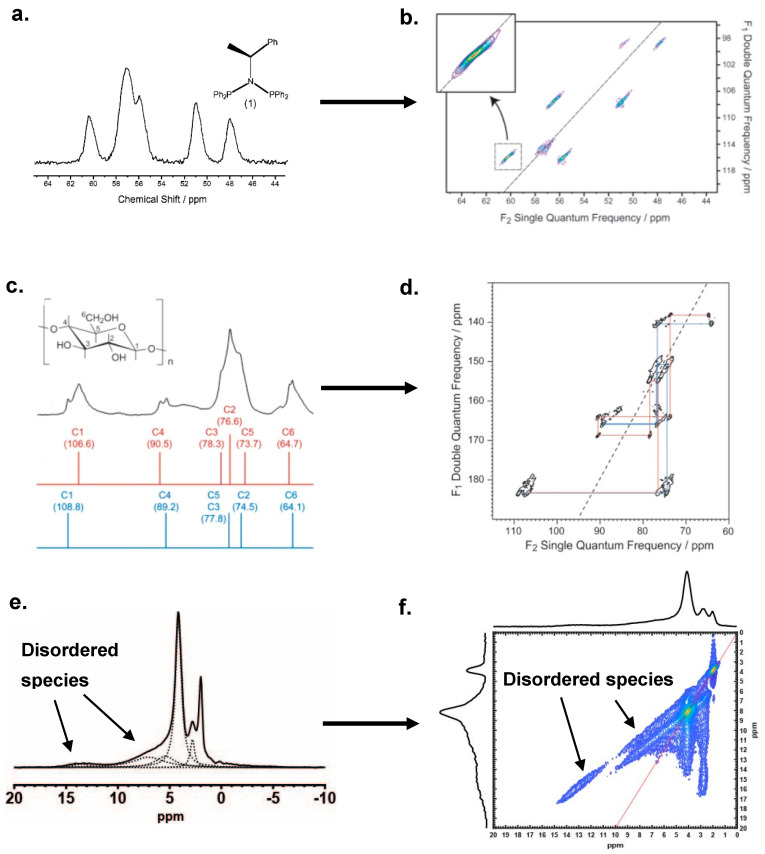
Typical types of disorder patterns illustrated by 1D MAS and 2D MAS DQ-SQ spectra in three materials, which are 1D (**a**) and 2D INADEQUATE (**b**) ^31^P NMR for *N*,*N*-bis(diphenylphosphino)-*N*-((S)-R-methylbenzyl)amine**;** 1D (**c**) and 2D INADEQUATE (**d**) ^13^C NMR for 10% carbon-13 labeled cellulose; 1D (**e**) and 2D dipolar-based DQ-SQ (**f**) ^1^H NMR for dehydrated zeolite catalyst HZSM-5. (**e**) was acquired at 9.4 T, at 10 kHz spinning frequency. (**a**–**d**) are reprinted (adapted) with permission from Ref. [13]. Copyright (2020) American Chemical Society. (**f**) is reprinted (adapted) with permission from Ref. [8]. Copyright (2020) American Chemical Society.

In a DQ–SQ NMR spectrum, the correlation is observed at the double-quantum frequency shown by the summed chemical shifts of coupled pairs in the single-quantum dimension. For example, for a correlated pair whose chemical shifts are δ_1_ and δ_2_ in the single-quantum dimension (F_2_), their correlation will be observed as two cross peaks horizontally aligned at δ_DQ_ = δ_1_ + δ_2_ in the double-quantum dimension (F_1_). Since the magnetization evolves at twice the isotropic frequency in the double-quantum dimension, a diagonal can be drawn with slope F_1_/F_2_ = 2 to assist in the interpretation of the spectrum. Inhomogeneous broadenings often appear as “stretched” ridges due to the dispersion of chemical shift, as illustrated previously in Figure 3d. The shapes and slopes of the ridges are important features since they correspond to changes of the local environment of the paired spins. Figure 4 shows a few typical lineshapes in DQ–SQ correlation NMR spectra caused by disorder. Figure 4a,c,e is the 1D MAS spectra of three different materials, namely, ^31^P NMR for *N*,*N*-bis(diphenylphosphino)-*N*-((S)-R-methylbenzyl) amine [13], ^13^C NMR for 10% carbon-13 labeled cellulose extracted from wood [13] and ^1^H NMR for dehydrated zeolite catalyst HZSM-5 [8]. Figure 4b,d,f are their associated 2D DQ–SQ spectra. Notably, the resolutions in these 1D spectra were substantially degraded by broadening and their 2D spectra contained several stretched cross peaks, which unambiguously indicate that the broadenings were inhomogeneous but not homogeneous. The latter typically yields circular shapes in 2D correlation spectra [51].

In the ^31^P DQ–SQ spectrum acquired by the INADEQUATE (Incredible Natural Abundance DoublE QUAntum Transfer Experiment) method, as shown in Figure 4b, three coupled pairs are observed at 99, 108 and 116 ppm in the double-quantum (F_1_) dimension. The two cross peaks in each pair are all stretched in the same direction, i.e., parallel to the diagonal with slope F_1_/F_2_ = 2. The “stretch effect” is caused by chemical shift distribution due to local field distribution. The reason that both peaks are elongated concomitantly is because the local fields of the paired atoms change identically, which is believed to be caused by a symmetrical structural disorder in this case [13,51]. However, anisotropic bulk magnetic susceptibility (ABMS) can also result in the similar “symmetric stretch” to the cross peaks, thus should always be carefully considered or ruled out when analyzing disordered structures. A more complex example is presented in the ^13^C spectra for the cellulose sample shown in Figure 4c,d. Note the remarkable resolution improvement and the rich correlation features of the 2D DQ–SQ (INADEQUATE) spectrum as compared to the 1D spectrum. These advantages of the 2D spectrum enable one to extrapolate the disorder information via the appearance of the cross peaks, particularly from the shapes and slopes. As a general observation, some cross peaks were correlated in the same direction (parallel to the diagonal), such as peaks at ca. F_1_ = 150 ppm, however, some were correlated in the reversed direction, such as the peaks at ca. F_1_ = 140 ppm. The peaks at ca. F_1_ = 165 and 180 ppm show even richer features. The authors Cadars et al. have proposed a model to systematically address the appearances for all correlated peaks, based on the population distribution of individual chemical shift components in the coupled spins [51]. As a concept adapted from their work, *y* = *kx* can be used as a convenient expression to address the peak features (particularly the slopes), where *k* is the virtual linewidth ratio between the correlated peaks, and *y* and *x* are the slopes for each of peaks. For instance, the peak pairs at F_1_ = 140 and 150 ppm can be expressed as *y* = −*0.25x* (negative sign indicate opposite direction) and *y* = *x*, respectively [51]. More complex situations such as the “horse shoe”-shaped cross peaks at F_1_ = 180 and the “arbitrarily”-shaped cross peaks at 165 ppm would require a comprehensive analysis with the model. Without examining the details of the model, the shapes can still be reasonably understood by the *k* value, i.e., both complicated shapes arise from overlapped cross peaks with different *k* values. This model provided by Cadars et al. may cover most lineshapes in the DQ–SQ spectrum, but a special case is worth mentioning, as shown in Figure 4f, which is a 2D DQ–SQ ^1^H MAS NMR spectrum for a dehydrated zeolite HZSM-5 recently reported by Chen et al. [8]. In this case, the lineshape corresponds to a system where significantly disordered species are coupled to non-disordered species. The narrow peaks at 2.8 and 4.2 ppm (non-disordered) are both coupled to the broad-line ranging from 5 to 10 ppm (disordered) as shown in the deconvoluted spectrum in Figure 4e, resulting in straight vertical ridges at both 2.8 and 4.2 ppm and two narrow ridges on the other side of the diagonal. The slopes of the latter ridges, however, are not parallel to the diagonal at F_1_/F_2_ = 2, but appear with the value of F_1_/F_2_ = 1, which is reasonable because with the chemical shifts of individual components within 5–10 ppm increasing along the F_1_ dimension, the 2.8 and 4.2 ppm peaks remained constant. Consequently, the slope was fixed to 1 due to the basic rule δ_DQ_ = δ_1_ + δ_2_. Note that the discussion above was focused on the lineshape analysis and not on the detailed structural implications. Although the lineshape analysis discussed here was limited to specific materials, it is clear that rich information about disordered structures can be extracted from 2D NMR spectroscopy, which could find important applications in a wide range of systems.

## 3. NMR Detection of Quadrupolar Nuclei in Disordered Environments

Solid-state NMR analysis of quadrupolar nuclei is more complex than spin-1/2 nuclei due to the existence of quadrupolar coupling, which can only be partially averaged out by magic-angle spinning with the remaining contribution typically much larger than CSA, dipolar coupling and the other line-broadening resources discussed previously. Disorder leads to distributions of both the chemical shift and EFG (electric field gradient). Therefore, the key to characterizing disorder in materials with quadrupolar nuclei involved materials is to disentangle the isotropic and quadrupolar interactions. The discussion in this section is focused on high field applications and MQMAS characterizations, which play important roles in quadrupolar NMR analysis.

### 3.1. NMR Spectroscopy of Quadrupolar Nuclei

#### 3.1.1. Quadrupolar Effect

**Figure 5:**
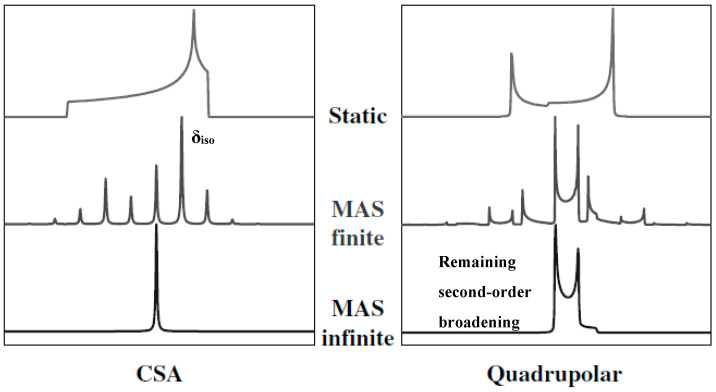
Simulated powder lineshapes in spectroscopy of spin-1/2 nuclei with only CSA (left) and spin-n/2 nuclei with only quadrupolar interaction (right) at static, finite MAS and infinite MAS conditions as denoted in the figure. With infinite MAS, CSA is completely removed in the left bottom figure, however a broad quadrupolar lineshape presents in the bottom right figure due the remaining second-order quadrupolar broadening. Adapted with permission from Ref. [55]. Copyright 2002, Wiley.

For quadrupolar nuclei, the NMR spectra are not only affected by CSA, dipolar- and J-couplings, but quadrupolar couplings that arise from the interaction between the electric quadrupole moment of the nucleus and the surrounding electric field gradient (EFG). Such quadrupolar couplings can range from a few kHz to several MHz or even a few GHz in magnitude [53,54]. The quadrupolar interaction as a perturbation of the Zeeman interaction can be expressed as: $H_{Q}^{full}=H_{Q}^{\left(1\right)}+H_{Q}^{\left(2\right)}+\ldots$, where $H_{Q}^{\left(1\right)}$ and $H_{Q}^{\left(2\right)}$ are referred to as the first-order and second-order quadrupolar interactions, respectively. The third-order terms are only necessary in uncommon occasions. For half-integer quadrupolar nuclei, which are primarily focused in review, the quadrupolar perturbation of the Zeeman interaction gives rises to central transition (CT) and satellite transitions (STs) in the NMR spectrum. However, the transitions are often significantly broadened in spectra acquired on powder samples. It is often only the relatively narrower CT that can be observed, being unaffected by the quadrupolar interaction to the first-order [18,20,53]. The detailed expression of the perturbation theory can be found in the book written by R. Wasylishen and S Ashrook et al. in Ref. [53]. The CT, however, is still affected by the second-order interaction, which contains high-rank anisotropic terms that are not completely averaged by magic-angle spinning, hence resulting in the remaining quadrupolar broadening to the spectrum. In a spinning sample at the axis of *β*, the time averaged NMR frequencies of the second-order interaction on the CT can be expressed as,

(2) $$\omega_{-1/2↔1/2}=\frac{\omega_{Q}^{2}}{\omega_{L}}\left[S\left(S+1\right)-3/4\right]\left[A_{0}+8A_{2}\left(\theta ,\varphi \right)P_{2}\left(cos\beta \right)+18A_{4}\left(\theta ,\varphi \right)P_{4}\left(cos\beta \right)\right]$$

where *S* is the spin quantum number, *ω_Q_* is the quadrupolar coupling constant, *ω_L_* is the Larmor frequency, *A*_0_ is a constant proportional to the isotropic quadrupolar shift, *A*_2_(*θ*,*φ*) and *A*_4_(*θ*,*φ*) are the orientation-dependent terms responsible for the anisotropic lineshape in the powdered spectrum and *P*_2_ and *P*_4_ are the second- and fourth-order Legéndre polynomials [18]. Under MAS where the spinning angle *β* = 54.74°, the second-rank term *P*_2_(*cosβ*) vanishes, however the fourth-rank term *P*_4_(*cosβ*) survives. It will require the sample to be spinning at a second angle *β* = 30.56° or 70.12° simultaneously to completely remove both *A*_2_(*θ*,*φ*)*P*_2_(*cosβ*) and *A*_4_(*θ*,*φ*)*P*_4_(*cosβ*), i.e., the anisotropic broadening terms [15,18,53]. The MAS effects on the powder lineshapes of both spin-1/2 (with only CSA simulated) and the CT of spin-n/2 nuclei (with only quadrupolar interaction simulated) are illustrated in Figure 5 as denoted, where finite and infinite spinning rates are applied [55]. As shown in the left figure, MAS broke the CSA pattern into an isotropic peak and spinning sidebands with finite spinning rate and totally removed the sidebands with the spinning rate increased to infinite. However, as shown in the right figure, the quadrupolar pattern was never completely eliminated even at the infinite spinning rate, due to the remaining fourth-rank term discussed above. The resulted CT peak under MAS contained the anisotropic lineshape whose width and shape were determined by the quadrupolar coupling constant C_Q_ and quadrupolar asymmetry parameter η_Q_, respectively. In the specific case in Figure 5 (right), the lineshape corresponded to η_Q_ = 0. Since quadrupolar broadening masks other interactions in the smaller scales, it needs to be reduced, removed or separated for further analysis to the spectrum. The second-order quadrupolar broadening is inversely proportional to the external magnetic field B_0_ in Hz and to B_0_^2^ in ppm, meaning increasing the field strength by a factor of 2 will narrow the line-width by a factor of 4 in ppm. Therefore, one obvious method to enhance the resolution for quadrupolar nuclei is to raise the field strength. Another important method is to employ two-dimensional methods such as 2D multiple-quantum magic angle spinning (MQMAS) [17,18] or satellite-transition magic angle spinning (STMAS) [19,20] experiments, where the second-order quadrupolar broadening is majorly removed by evolving the magnetization via multiple-quantum or satellite transitions in the indirect dimension.

#### 3.1.2. Resolving Quadrupolar Species with Higher Fields and/or MQMAS Method

**Figure 6:**
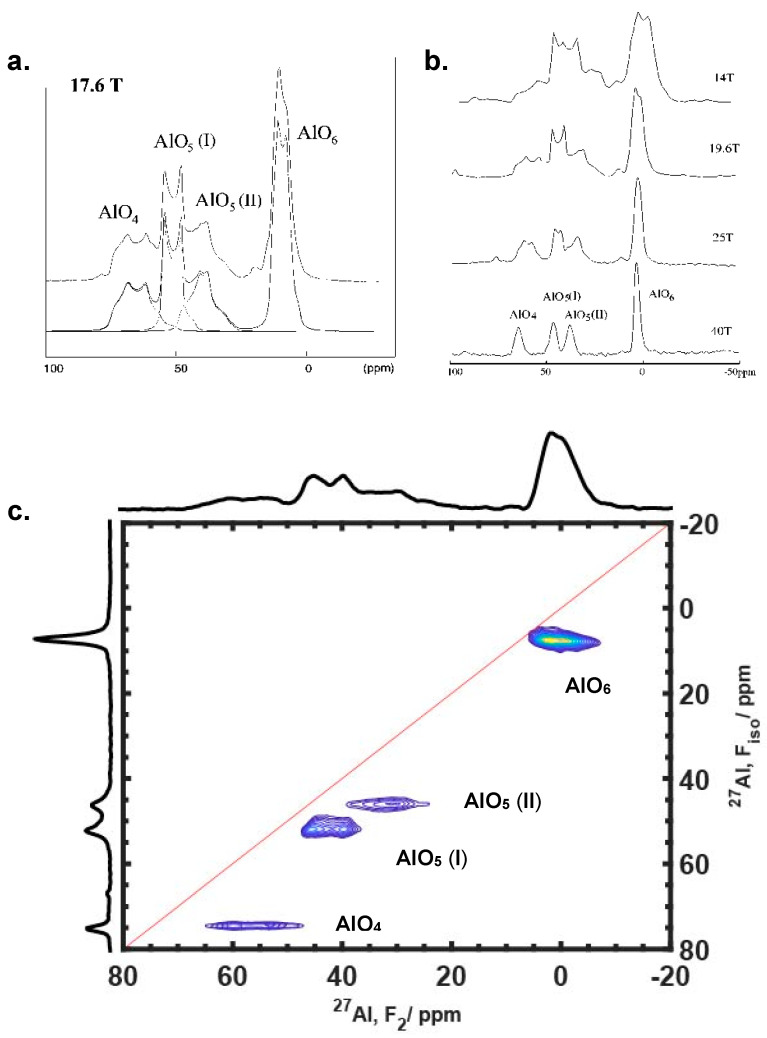
^27^Al MAS NMR of A9B2 (9Al_2_O_3_·2B_2_O_3_) acquired at 17.6 T showing the deconvolution of four sites (**a**) and, at four different fields 14, 19.6, 25 and 40 T showing field-dependent resolution enhancement (**b**). All four sites are completely resolved at 40 T where quadrupolar broadening is majorly removed. The peaks are also resolved in the 2D MQMAS spectrum at 19.6 T as shown in (**c**). (**a**,**b**) are reprinted (adapted) with permission from Ref. [22]. Copyright (2002) American Chemical Society. (**c**) was acquired at 19.6 T at National High Magnetic Field Laboratory (NHMFL).

Performing experiments at high fields and using the MQMAS method are two common means to study quadrupolar nuclei. ^27^Al MAS NMR spectra of A9B2 (9Al_2_O_3_·2B_2_O_3_), a crystalline sample containing four distinct Al sites, denoted as AlO_4_, AlO_5_ (I), AlO_5_ (II) and AlO_6_, were acquired at multiple magnetic fields up to 40 T by Gan [22], and are presented in Figure 6b to illustrate the field-strength effects on the quadrupolar line shape and width. The deconvolution of the four sites is shown in the spectrum acquired at 17.6 T in Figure 6a, where broad quadrupolar patterns dominate the lines and result in significant overlaps. Dramatic line-narrowing is observed in the spectra acquired with field strength increased, at 14, 19.6, 25 and 40 T, respectively. Notably, the quadrupolar pattern disappears at 40 T, leaving all peaks resolved completely. Though raising the field strength is a neat and straightforward method, it is not always conveniently accessible. As a complementary method, MQMAS, currently the most popular experiment for quadrupolar nuclei analysis [11,56], offers the capability of resolving quadrupolar sites at lower fields. Figure 6c shows a 2D MQMAS spectrum of A9B2, which was acquired at 19.6 T and performed with isotropic shearing [17,57]. The shearing process is normally necessary for interpreting the MQMAS spectrum because the magnetization evolves at different frequencies (due to multiple-quantum transition) in the indirect dimension, unless the split-t1 method is employed during the experiment [58]. In the isotropically sheared spectrum, theAl sites appears as horizontal ridges, with the direct F_2_ dimension broadened by the second-order quadrupolar coupling and the indirect F_iso_ (or F_1_) dimension separated at δ_F1_ = δ_iso_ − 10/17δ_qis_, respectively, where δ_iso_ and δ_qis_ are the isotropic chemical shift and quadrupole induced shift [21,57]. Note that the F_2_ projection nearly reproduces the 1D spectrum shown in the 19.6 T trace in Figure 6b. The F_iso_ projection, however, shows completely resolved peaks of all four Al sites due to the removal of the second-order quadrupolar broadening.

### 3.2. Disorder Observed by MQMAS Spectroscopy

Due to its capability of separating isotropic and quadrupolar features, the MQMAS method is a promising approach to disentangling the distributions of the isotropic chemical shift δ_iso_ and EFG, which can be induced by disorder or crystalline inequivalent sites. The distribution of EFG results in distributions of C_Q_, δ_qis_ and η_Q_, specifically. An inspection of all the parameters that affect the lineshapes in MQMAS spectroscopy revealed three essential parameters, namely, the “distribution of δ_iso_”, “magnitude of C_Q_” and “distribution of C_Q_”. Common features of the MQMAS lineshapes are summarized and modeled in six scenarios in Table 1, in terms of the contribution of each parameter, denoted “P” for prominent and “N” for non-prominent. It is important to notice that the non-prominent “magnitude of C_Q_” does not include C_Q_ = 0, because the multiple-quantum transitions will not be observed if there is no quadrupolar interactions. It merely represents the case where C_Q_ is very small and/or the field strength is large so that quadrupolar broadening is minor or overwhelmed by other interactions in the spectrum. The quadrupole induced shift δ_qis_ is surely an important parameter, but not included in Table 1 for the sake of simplicity, as it is a function of C_Q_ by itself.

**Figure 7:**
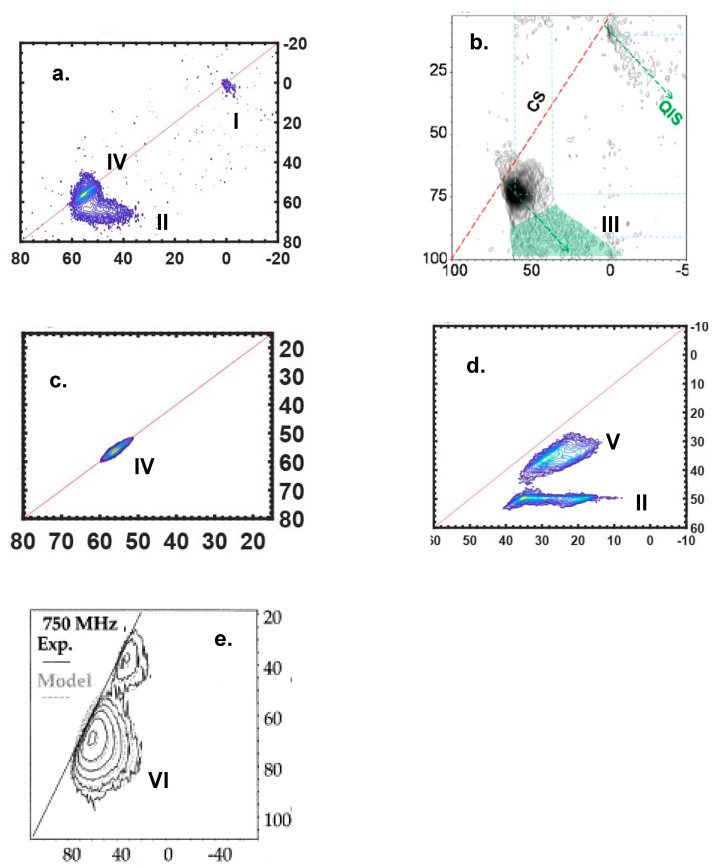
Experimental spectra showing sheared MQMAS spectra of the six scenario lineshapes described in Table 1, with the indication of chemical shift (CS) and/or quadrupole induced shift (QIS) lines. Projections are not shown as the focus is on the lineshape analysis. The detailed information of all figures are: (**a**) ^27^Al MQMAS for hydrated zeolite HZSM-5 at 14.1 T; (**b**) ^27^Al MQMAS of zeolite HUSY; (**c**) ^27^Al MQMAS for the same hydrated zeolite HZSM-5 in (**a**) but at 35.2 T; (**d**) ^17^O MQMAS for hydrated zeolite HZSM-5 at 18.8 T and (**e**) ^27^Al MQMAS of CaO-Al_2_O_3_-SiO_2_ glass at 17.6 T. (**a**) and (**c**) are reprinted (adapted) with permission from Ref. [8]. Copyright (2020) American Chemical Society. (**b**) is reprinted from Ref. [59], Copyright (2010), with permission from Elsevier. (**d**) was acquired at 18.8 T at National High Magnetic Field Laboratory. (**e**) is reprinted from Ref. [2], Copyright (2004), with permission from Elsevier.

Figure 7 shows a few representative experimental results selected from the literature [2,8,59] to illustrate the scenarios summarized in Table 1. As the specific information for each material and experiment is listed in detail in the caption of Figure 7, we focused here instead on the lineshape analysis. The scenarios in Table 1 and their corresponding peaks in Figure 7 are labeled with the same Roman numerals. The details of MQMAS theory and its fundamentals are described explicitly in the literature [17,18,57,58,60], but we described here a few empirical trends in the appearance of the lineshapes in i-sheared MQMAS spectroscopy, in order to point out typical connections between the spectroscopic features and the changes to the NMR parameters. Similar trends have been previously illustrated by Amoureux and Pruski [61]. The diagonal, or the chemical shift line, which is drawn in each spectrum in Figure 7, provides the most important guidance for addressing the identity of each resonance. A universal trend can be found in the MQMAS spectrum, as a function of the value of C_Q_. For small values of C_Q_, the 2D peak appears closer to the chemical shift line; for larger values of C_Q_, the peak moves further from the chemical shift line and the lineshape appears broader in the F_2_ dimension. The center of gravity of a given resonance along the F_2_ dimension is determined by δ_center_ = δ_iso_ + δ_qis_, where the quadrupole induced shift δ_qis_ has a negative sign and its absolute value is proportional to C_Q_. The reason that the peak moves away from the chemical shift line is that the shift in the isotropic dimension (F_1_ or F_iso_) is defined by δ_F1_ = δ_iso_ − 10/17δ_qis_. In other words, if C_Q_ increases, |δ_qis_| increases so that δ_F1_ becomes larger. In terms of the feature to the spectrum, increasing C_Q_ will generally elongate the width of the horizontal ridge and move it away (downward) from the chemical shift line, and importantly, it will follow the QIS (quadrupole induced shift) line. These empirical trends regarding the behavior of the MQMAS spectral pattern, with respect to the NMR parameters, will assist in the interpretation of the different features illustrated in spectra Figure 7. Although the 2D spectral patterns in Figure 7 arise from species with various C_Q_, at variable fields and with multiple features, they can still be categorized into the scenarios listed in Table 1, as discussed below:

Scenarios “I” and “IV” represent the cases where the quadrupolar effect is non-prominent or even negligible, which is often observed for small C_Q_ species. Given that the second-order quadrupolar broadening was inversely proportional to B_0_^2^ in ppm, the quadrupolar effect on the lineshape will eventually “vanish” at some point with increasing magnetic field strength, examples of which are shown in “I” in Figure 7a and “IV” in both 7a and 7c. Note the strength of a “vanishing field” varies from case to case depending on the C_Q_ of the observed species. In this scenario, the δ_iso_ distribution dominated the lineshape and could be directly estimated from the linewidth (in either dimension) of the 2D ridge. “I” in Figure 7a shows a nearly round-shaped “cross peak”, indicating the absence of chemical shift distribution. “IV” in Figure 7a,c appeared as a stretched narrow ridges along the chemical shift diagonal similar to the “stretch effect” for spin-1/2 nuclei, where the chemical shift distribution could be estimated as ca. 7 ppm.

In scenario “II”, when the field was not strong enough to remove the quadrupolar effect, the second-order quadrupolar broadening becme important and started to affect the spectrum. For a nuclear site without a chemical shift distribution but only quadrupolar broadening, e.g., a single crystalline site, the lineshape appeared as a narrow horizontal ridge, as previously shown for the A9B2 sites in Figure 6 and “II” in Figure 7d. This type of horizontal ridge could also be broadened in the isotropic dimension. For instance, another horizontal peak “II” in Figure 7a was broadened in the isotropic dimension as well, probably due to a combination of small degrees of both a chemical shift distribution and C_Q_ distribution (because C_Q_ distribution results in δ_qis_ distribution, which is encoded in the isotropic dimension).

In scenario “III”, where the resonance arose from species possessing a distribution of C_Q_, while the quadrupolar broadening remained prominent, a triangle-shaped pattern will form, as illustrated in “III” in Figure 7b, shaded in green color. The shape can be understood by referring to the empirical trends discussed above: Distinct sites with different C_Q_ values appeared as individual horizontal ridges and are distributed along the QIS line with increasing linewidths, and their superposition will result in a triangle pattern. When the resolution diminished in the F_iso_ dimension, an overall triangle-shaped pattern would be observed, as shown in Figure 7b. This effect often occurred in crystalline materials with inequivalent crystalline sites, such as zeolites.

In scenario “V”, when C_Q_ was large enough to induce prominent broadening but had a non-prominent sized distribution, while δ_iso_ had a large sized distribution, the total resonance consisted of a superposition of horizontal ridges with a similar linewidth, visualized as a broad ridge parallel to the chemical shift line, as shown in “V” in Figure 7d.

Finally, scenario “VI” describes the case when the effects of all three parameters listed in Table 1 are prominent, which often occurred in totally disordered materials such as glasses, and the features of the lineshape in this case become challenging to directly interpret, as shown in Figure 7e. However, because the distributions of the parameters were continuous, the spectrum can often be fitted with the Czjzek model (see below) to obtain averaged NMR parameters.

### 3.3. Fitting Disordered Lineshapes with the Czjzek Model

**Figure 8:**
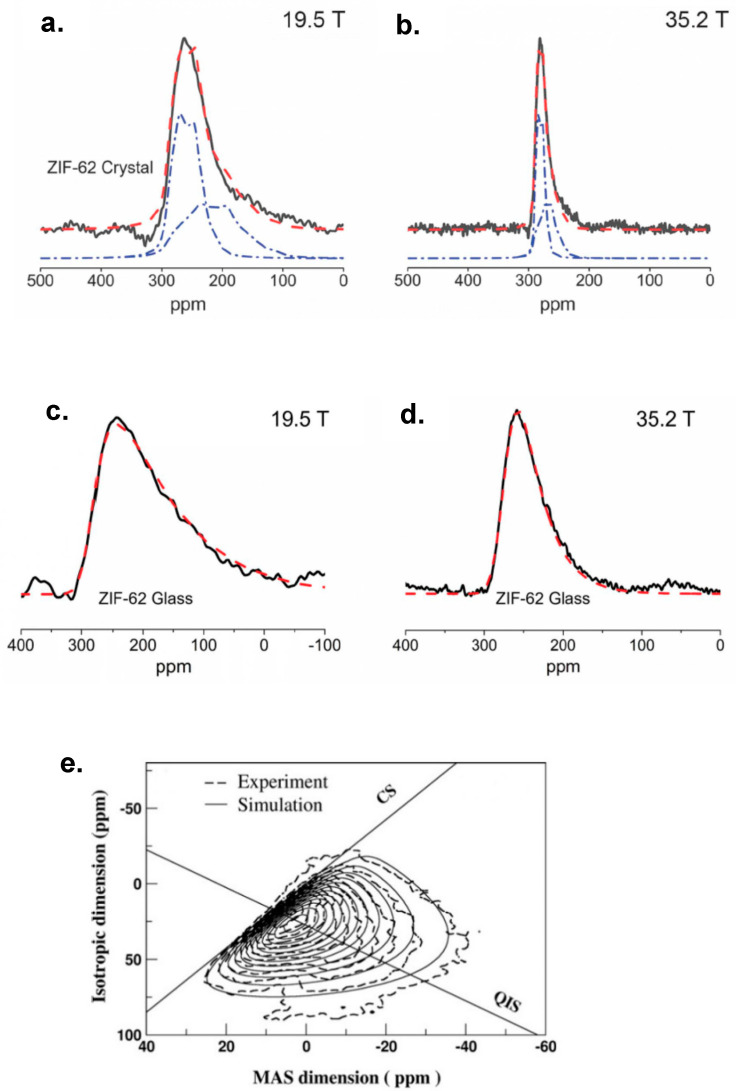
Fittings of ^67^Zn 1D MAS NMR acquired for a metal-organic framework material ZIF-62 in its crystalline (**a**,**b**) and glass (**c**,**d**) forms, at two different fields 19.6 and 35.2 T as indicated in the figure. Blue dashed lines in (**a**,**b**) are quadrupolar fitted crystalline sites and red dashed lines are the sum of the fittings. Red dashed lines in (**c**,**d**) are disorder fittings resulted from Czjzek model. (**e**) shows a representative Czjzek fitting for 2D MQMAS spectrum acquired for ^23^Na of a disordered sodium silicate glass material. (**a**–**d**) are adapted from Ref. [1]. Reprinted with permission from AAAS. (**e**) is reprinted from Ref. [66], Copyright (2011), with permission from Elsevier.

Spectra with distinct quadrupolar sites can usually be conveniently deconvoluted with quadrupolar parameters, but this is not the case for totally disordered amorphous materials in which C_Q_ and δ_iso_ are randomly distributed. However, fitting the spectrum using the Czjzek model [62,63] can provide a way to obtain the root mean-square quadrupolar product $\sqrt{C_{Q}^{2}}$ by treating C_Q_ and δ_iso_ as normal (Gaussian) distributions [64,65]. Figure 8a,b present two ^67^Zn MAS NMR spectra of a crystalline metal-organic framework sample ZIF-62, acquired at 19.6 and 35.2 T, respectively, where two crystalline sites were well deconvoluted in both spectra. Note the crystalline linewidths were narrowed to almost a quarter as the field is nearly doubled. By melting and quenching, the crystalline sample was turned into a disordered glass form, as the crystallization process was prevented by quenching. The consequent spectra for the melt-quenched ZIF-62 sample are shown in Figure 8c,d. By losing the long-range periodicity in the structure, quadrupolar deconvolution failed since no distinguishable sites existed. In this case, fitting with the Czjzek model could provide satisfying results, as shown in the dashed red lines in Figure 8c,d, and the root mean-square quadrupolar product $\sqrt{C_{Q}^{2}}$ could be obtained [1]. In general, a random distribution of C_Q_ will result in lineshape tailings toward high fields (smaller chemical shifts), as clearly shown in Figure 8c,d. This behavior is reasonable because the second-order quadrupolar broadening always resulted in high field shifts, and therefore, the sum of a random distribution of those shifts will result in a unidirectional broadening toward the high field [64]. The Czjzek model can also be applied to fit the lineshapes arising from totally disordered species in 2D MQMAS spectra, as shown in Figure 8e, where the dashed lines were experimental and the solid lines were the simulated spectrum [66]. Fitting procedures using the Czjzek model are now included in several pieces of academic software such as Dmfit [55] developed by Massiot et al. and ssNake [65] developed by Kentgens et al.

### 3.4. Practical Problems and the Strategy for Disorder Characterizations

#### 3.4.1. Weak Sensitivity and Broad-Line Problems

Given that the NMR sensitivity for a quadrupolar nucleus is a function of γ (gyromagnetic ratio), natural abundance, quadrupole moment and field strength, and that low-γ, low natural abundance and high quadrupole moment are not unusual, low sensitivity and broad-line issues are common problems. Two-dimensional experiments can be more challenging because (a) 2D experiments require much longer time due to the repetitions in the second dimension; (b) the sensitivity drops significantly from 1D to 2D acquisition. For instance, the 3QMAS (triple-quantrum magic-angle spinning) efficiency, although reaches as high as 30% for spin systems with I = 5/2 and 55% for I = 3/2 in theory [67,68], is usually below 20% in practice, and can be as low as a few percent for resonances with large C_Q_’s; and (c) broad-line widths hinders uniform and efficient excitation of pulses and cause lineshape distortions to the spectrum [69]. Increasing the external field strength is the most obvious method to resolve the low sensitivity problem. It also helps with the broad-line problem since the quadrupolar broadening reduces dramatically as field strength is increased. For a comparison, doubling the field strength will narrow a quadrupolar broadening by a factor of 4 and may increase the quadrupolar nucleus sensitivity by a factor of 20, or by a factor of 400 in time savings [70]. Such boosts can immediately broaden the range of feasible NMR experiments. Another option is to employ sequences designed for signal enhancement, such as ST (satellite transition) population transfer based methods, which are approached by sweeping the satellite transitions with adiabatic WURST (wideband, uniform rate and smooth truncation) [71] or DFS (double-frequency sweep) [72] shaped pulses. The employment of these adiabatic pulses can increase the polarization of the central transition and provide signal enhancement by a factor of (S+1/2) to 2S, or by a factor up to 2-3 in practice. The QCPMG (quadrupolar Carr–Purcell–Meiboom–Gill) method, adapted from CPMG sequence [73,74], which refocuses magnetization repetitively, resulting in a set of spikelets resembling the broad powder pattern lineshapes upon Fourier transform, can increase the sensitivity by a large factor, sometimes greater than 10 [24,25]. ST-population transfer and QCPMG enhancement sequences can also be incorporated into regularly used methods to achieve optimum sensitivity [30,75]. For the broad-line issue where MAS speed becomes a limitation as the anisotropic linewidth exceeds the spinning frequency, sideband removal sequences such as QMAT (quadrupolar magic-angle turning) [28] and QPASS (quadrupolar phase-adjusted sideband separation) [29] can be applied to separate the modulated and averaged anisotropic parts into two dimensions [76]. However, the sideband separation methods are achieved by 2D experiments, and are therefore, only available for materials with high sensitivities.

#### 3.4.2. General Strategy for Disorder Characterization

Although MQMAS and the Czjzek fitting method can provide promising information about disordered structures, another method worth mentioning to obtain the degree of chemical shift disorder is the variable-field experiment. Since the portion of line-broadening (in ppm) contributed by δ_iso_ distribution is constant, while the portion from quadrupolar broadening is inversely proportional to B_0_^2^, the overall linewidth for a spectrum with different weighting fractions of these two types of broadenings will be narrowed differently upon increasing the field strength. For example, the linewidths were narrowed more dramatically from Figure 8a to Figure 8b than from Figure 8c to Figure 8d, as the latter had a significant disorder. Specifically, if the portion of the isotropic chemical shift distribution (in ppm) is denoted “C”, and the portion of quadrupolar broadening is denoted “Q”, both of them can be obtained by linear fitting using the equation: FWHM = C + Q/B_0_^2^ [8]. However, this method only provides a semiquantitative analysis due to the uncertainty of the linewidth measurement.

Considering both the isotropic chemical shift distribution and the quadrupolar broadening, characterizing structural disorder in quadrupolar NMR is a debate between selecting the most appropriate field strengths and NMR experiments. High fields favor the sensitivity and linewidth (i.e., resolution), however, too high of a field will diminish the quadrupolar effects, thus diminishing the disorder information encoded in the EFG. A good example is the ^27^Al MQMAS NMR spectrum acquired for hydrated zeolite at 35.2 T in Figure 7c, where the C_Q_ of the aluminum species are 1–2 MHz [8]. For the same sample, more quadrupolar features can be found in the spectrum acquired at 14.1 T, as shown in Figure 7a. Hence, the highest field is not always appropriate, and instead the field strength should be carefully selected. In the high field or low C_Q_ cases where quadrupolar effects are non-prominent, MQMAS, DQ–SQ and HETCOR experiments should be favorably considered. In cases where quadrupolar broadening is prominent, the MQMAS spectra can be analyzed by the model in Table 1 and Figure 7. The DQ–SQ and HETCOR spectroscopy, although expected to be more complicated is this case, can still provide valuable information [59,77,78,79]. In cases where the quadrupolar interaction is too large and/or sensitivity is too low so that 2D methods are not suitable, variable-field 1D experiments are the most reasonable options. Czjzek fitting and variable-field linewidth fitting can be performed in certain situations, noting the former only fits for totally disordered materials and the latter can be applied to all materials. If the sensitivity is good but the linewidth exceeds the spinning frequency, sideband separation methods can be applied, followed by Czjzek fitting and variable-field linewidth fittings.

## 4. Conclusions

In this review, NMR lineshape analysis for solids was shown to be capable of extracting considerable information about structural disorder for both spin-1/2 and quadrupolar nuclei. As for spin-1/2 nuclei, the structural disorder created local field dispersion, and thereby caused a chemical shift dispersion, which was visualized as inhomogeneous broadening in 1D and stretched or distorted cross correlation peaks in 2D spectroscopy. The effects of the disorder on lineshapes were discussed in detail in examples of 2D DQ–SQ correlation spectroscopy. For quadrupolar nuclei, the remaining second-order quadrupolar broadening at the MAS condition complicated the spectrum. Disorder information could be encoded in lineshapes affected by both a chemical shift and EFG distribution. MQMAS, the most popular method for separating quadrupolar broadening and isotropic chemical shifts, was shown to provide disorder information by a lineshape analysis, through the use of an established model. We have also shown that the field strength plays an important role in characterizing disorder structures in quadrupolar NMR methods. Though we have restricted our focus to specific examples of lineshape analysis in this review, the strategies and methods presented are general and can have broad experimental applications.

## Figures and Tables

**Table 1:** Six scenarios of regular lineshapes in MQMAS spectrum determined by combinations of “distribution of δ_iso_”, “distribution of C_Q_” and “magnitude of C_Q_”. “N” stands for non-prominent (does not mean “not exist”), “P” stands for prominent. The key feature of each pattern is also commented. Examples of real spectra are shown in Figure 7.

Scenarios	δ_iso_	C_Q_		Features of Lineshape
	Distribution	Magnitude	Distribution	
I	N (Non-prominent)	N	N	Round
II	N	P	N	Horizontal ridge
III	N	P	P	Triangle
IV	P (Prominent)	P	N	Ridge along diagonal
V	P	P	N	Wide ridge parallel to diagonal
VI	P	P	P	Need fitting for analysis
